# Correlation Analysis and Prediction Model of Thermal Protection Performance of Aramid 1414 Fabric

**DOI:** 10.3390/polym15051188

**Published:** 2023-02-27

**Authors:** Zhemin Zhang, Jinzhong Zhang, Xiangyu Ye, Keai Ma, Haihang Li

**Affiliations:** 1 College of Quality and Safety Engineering, China Jiliang University, Hangzhou 310018, China; 2 Wenzhou Darong Textile Instrument Co., Ltd., Wenzhou 325016, China; 3 Zhejiang Light Industrial Products Inspection and Research Institute, Hangzhou 310000, China; 4 Key Laboratory of Furniture Inspection Technology of Zhejiang Province, Hangzhou 310018, China

**Keywords:** Aramid 1414, thermal protection performance, air gap, correlation analysis

## Abstract

The thermal protection performance of fire suit is vital to the safety of firefighters. Using certain physical properties of fabrics to evaluate their thermal protection performance speeds up the process. This work aims to develop a TPP value prediction model that can be easily applied. Five properties of three types of Aramid 1414 made of the same material were tested, and the relationships between the physical properties of Aramid 1414 and its thermal protection performance (TPP value) were investigated. The results showed that the TPP value of the fabric had a positive correlation with grammage and air gap, and a negative correlation with the underfill factor. A stepwise regression analysis was used to solve the collinearity issue between the independent variables. Finally, a model for predicting TPP value by air gap and underfill factor was developed. The method adopted in this work reduced the number of independent variables in the prediction model, which is conducive to the application of the model.

## 1. Introduction

Firefighters are often exposed to various types of hazards when carrying out rescue missions, including flash fire, high temperature, and thermal radiation [1]. Thermal protective clothing is designed to mitigate burns and reduce the risk of injury or death when personnel are exposed to unpredictable heat [2]. As an important defense to protect firefighters in a fire scene, the thermal protection performance of firefighters’ clothing are vital to their lives. Direct test of thermal protection performance requires expensive equipment. It is well-known that the properties of the fabrics used in thermal protective clothing determine its performance under heat exposure. If certain properties of a fabric can be used to indirectly evaluate or predict its thermal protection performance, then tests can be reduced to speed up the evaluation process. This work is one of the hot spots in the study of the thermal protection performance of fabrics.

Factors affecting the thermal protection performance of fabrics are grammage, thickness, air permeability, and air gap. It has been found that the thermal protection performance of fabrics increased with the increase in thickness [3,4,5]. There was a positive correlation between grammage and thermal protection performance [5,6]. The thermal protection performance of fabrics decreased with increasing air permeability [6,7]. The presence of an air gap between the fire suit and the skin in both bench scale test and flame manikin test showed a great increase in the TPP value of the fabric [3,4,6]. The air gap is not uniformly distributed and the size of the air gap depends on the style and fitness of the garment with the body contour [8].

Several models have been developed to predict thermal protection performance. Ghalnxy et al. [9] developed a finite volume model to study the effect of fabric heat shrinkage on thermal protection performance, while considering a model of the air gap between clothing and skin. Su et al. [10] developed a heat and moisture transfer model based on partial differential equations considering the effects of phase change, absorption/desorption, and moisture on heat exchange. Mandal et al. [7,11] developed multiple linear regression (MLR) and artificial neural network (ANN) models for predicting the thermal protection and thermal-physiological comfort performance of fabrics. However, most of the previous works on multiple regression models have not considered reducing the number of independent variables, and little consideration has been given to collinearity issue among independent variables.

Existing regression prediction models generally include all the factors that affect the thermal protection performance in the model, which affects the application of the prediction model. The above problems would be better addressed if certain key factors could be identified to reduce the independent variables of the prediction model. After reviewing the role of the above influencing factors, it was seen that there may be some inherent relationship between parameters such as grammage, thickness, and air permeability. In general, the thicker and heavier the fabric, the less permeability it is. Therefore, a combined parameter (e.g., underfill factor) representing the performance of these parameters could be the key factor. On the other hand, there may be a collinearity issue between some parameters, i.e., one parameter can be represented by another. Therefore, we will attempt to introduce a combined parameter to reduce the number of independent variables in the prediction model.

This work first tested the thermal protection performance and physical properties of Aramid 1414. The relationships between the physical properties of the fabric and the air gap and the thermal protection performance were then analyzed. Grey correlation analysis was used to determine the influence degree of each factor on the thermal protection performance. A stepwise regression method was then used to eliminate the collinearity problem. And a multiple regression prediction model for thermal protection performance (TPP value) was finally established.

## 2. Materials and Methods

The TPP value and physical property test data of the fabrics are the prerequisites for this work. It can be speculated that the grammage, thickness, air gap, and underfill factor of a fabric may be the determining factors for its TPP value from the literature review in Section 1. Therefore, we obtained experimental data by means of measurement tools corresponding to these physical properties.

TPP value is most commonly used to assess the thermal protection performance of fabrics. In this work, the TPP value measurement was carried out in accordance with *Protective clothing—Thermal protective performance test method* [12]. The TPP values of the fabrics were measured using a thermal protection performance tester (model type: DR255).

**Figure 1:**
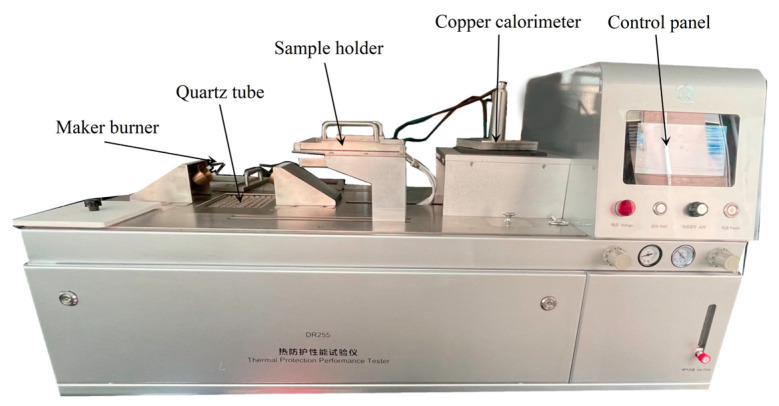
TPP test apparatus.

The TPP experimental setup (Figure 1) provides a combustion flame through two burners and a radiant heat source through a quartz lamp. A copper calorimeter test sensor is used to monitor the temperature instead of human skin. Different air gap sizes are simulated by setting metal spacers of different thicknesses on the sample holder (see Table 1, air gap range 0–19.2 mm). The total heat flux is set at 84 kW/m^2^. The TPP value is the total heat flux multiplied by the second-degree burn time [13,14]. The higher the TPP value, the better the thermal protection performance of the fabric.

In this work, three different types of Aramid 1414 (para-aramid) of the same material and with the same knitting method, were used for the experiments. Aramid 1414 has excellent properties such as ultra-high strength, high modulus, acid and alkali resistance, light weight and high temperature resistance [15,16]. It does not decompose or melt at 560 °C, so it has excellent high temperature resistance. Table 1 shows the physical properties of Aramid 1414 such as grammage, thickness, and underfill factor. Common physical parameters including grammage and thickness were measured in this work by conventional methods, which are not described here.

The underfill factor refers to the ratio of the individual loop length to the yarn diameter of a knitted fabric and is expressed as:

(1) $$\delta =\frac{L}{\mathrm{ND}}$$

where δ is the underfill factor, L is the total length of the coils of a 10 cm specimen (mm), and D is the diameter of the yarn (mm). In the experiment, the number of coils N of a 10 cm specimen was measured, and the length of the coils L of a 10 cm specimen was measured using the unraveling method. Five test results at different positions were averaged to calculate the underfill factor of the fabric.

## 3. Results and Discussion

### 3.1. Correlation Analysis between Fabric Properties and TPP Value

Correlation analysis analyzes two or more variables that may be correlated, so that the correlation degree between the variables can be measured. In order to identify the key physical properties that influence the thermal protection performance of Aramid 1414 fabrics, this work will first correlate each fabric property with the TPP value.

**Figure 2:**
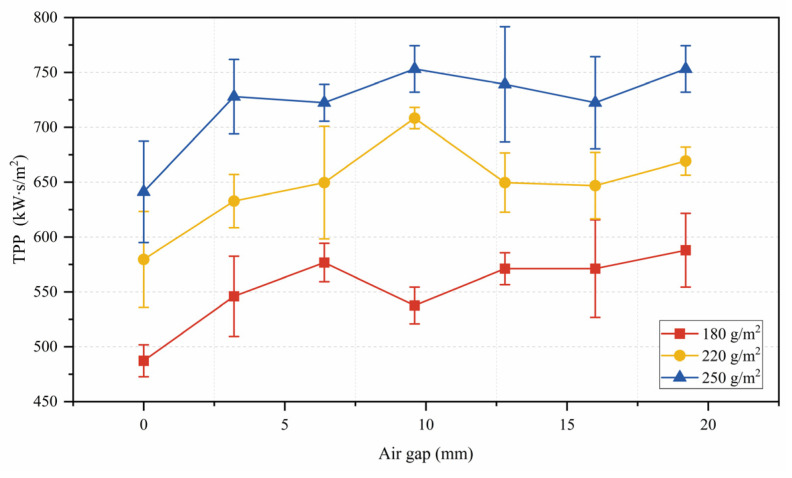
TPP values of different air gaps.

Table 2 and Figure 2 show the TPP values of the three types of Aramid 1414 at different air gaps. The TPP value for the different grammage fabrics all showed an overall increasing trend with increasing air gap. For Aramid 1414 with a grammage of 180 g/m^2^: (1) The TPP value showed an overall increasing trend when the air gap gradually increased from 0 mm to 6.4 mm. (2) The TPP value decreased with increasing air gap from 6.4 mm to 9.6 mm. (3) They increased steadily with increasing air gap from 9.6 mm to 19.2 mm. For Aramid 1414 with grammages of 220 g/m^2^ and 250 g/m^2^, the TPP value increased when the air gap increased from 0 mm to 9.6 mm, and then decreased with increasing air gap from 9.6 mm to 16 mm.

In the experimental results of this work, for fabrics of different grammages, the TPP value showed an inflection point at 6.4 mm or 9.6 mm air layer thickness. When the thickness of the air layer between the fabric and the skin is less than or equal to 6.4 mm, heat is transferred by conduction and radiation; when the thickness of the air layer is greater than 6.4 mm, convective heat transfer occurs [17,18] and the thermal insulation effect of the air gap diminishes [4]. This change in the heat transfer mechanism explains the results of this work, i.e., the TPP value increased steadily when the air gap was less than about 6.4 mm, while the TPP value stopped increasing when the air gap was greater than about 6.4 mm.

**Figure 3:**
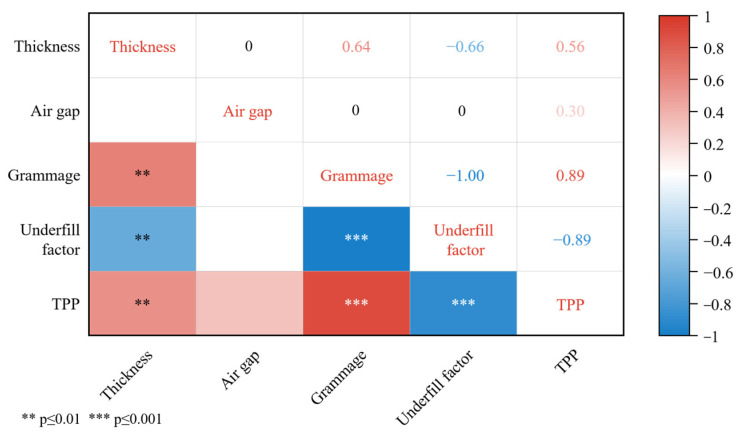
Heat map of correlation coefficients.

The physical properties of the fabric have an effect on its thermal protection performance, acting in a positive or negative role. The correlations between the grammage, air gap, underfill factor, thickness, and TPP value of the fabric were analyzed and the results are shown in Figure 3. It can be seen that the relationships between the four factors and the TPP value are as follows: (1) the correlation coefficient r between grammage and TPP value was 0.89, the significance probability *p* < 0.001. The latter indicated that the two parameters were significantly correlated at 0.001 level. Therefore, there was a significant positive correlation between grammage and TPP value, i.e., TPP value increased with increasing grammage. (2) The correlation between air gap and TPP value was r = 0.3 and was not significant. (3) The r = 0.56, *p* < 0.01 between thickness and TPP value indicated a positive correlation between these two parameters. (4) The r = −0.89, *p* < 0.001 between underfill factor and TPP value indicated a significant negative correlation between the two parameters. The TPP value of Aramid 1414 decreased as the underfill factor of the fabric increased. This is due to the fact that the larger the underfill factor, the more sparse the fabric structure is and the better the breathability is at a constant yarn diameter [19]. The more breathable a fabric is, the easier it is for heat to be transferred through the fabric, resulting in a decrease in the thermal protection of the fabric [6,7]. Therefore, a significant negative correlation was shown between the underfill factor and the TPP value.

**Figure 4:**
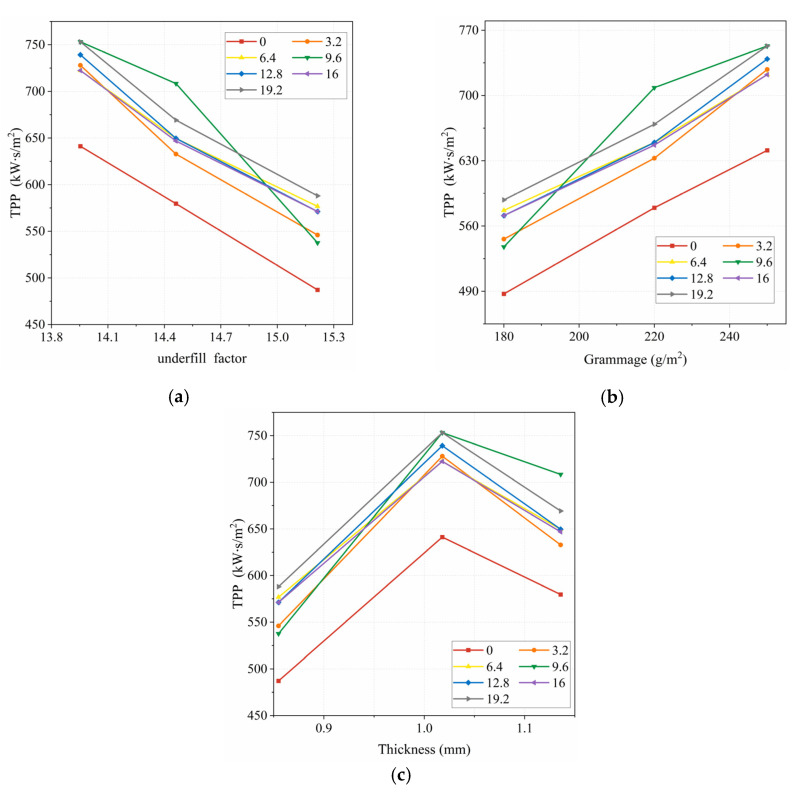
Relationship between underfill factor, grammage, thickness and TPP value: (**a**) TPP value and underfill factor; (**b**) TPP value and grammage; (**c**) TPP value and thickness.

Figure 4 shows the correlation between underfill factor, grammage, thickness, and TPP value for different air gaps. From Figure 4a,b, it can be seen that the TPP value decreased with an increasing underfill factor, and increased with an increasing grammage. As it can be seen from Figure 4c, the TPP value increased and then decreased with fabric thickness. This was due to the fact that the thickness of the fabric is related to the loop length, the bending depth scale value, the fabric organization, and the flat knitting machine gauge [20]. Therefore, the TPP value did not show a continuous increase with increasing fabric thickness in this work.

### 3.2. Analysis of Influencing Factors Based on Grey Correlation Analysis

Grey correlation analysis is a reliable and simple mathematical analysis method that uses the grey correlation grade to analyze the contribution degree of each sub-factor to the main factor [21]. It has no requirement for sample size and is computationally small and time-consuming. This work used grey correlation analysis to find the contribution degree of the influencing factors on the TPP value. The reference series is the TPP value (x_0_) and the comparison series (x_i_) are the grammage, gap, thickness, and underfill factor.

The units of the variables in the above series are different and the variables cannot be used directly for comparison. In order to eliminate the effect of different units, the variables need to be normalized. The most common methods of normalization are vector normalization and linear proportional transformation [22]. In this work, the min-max normalization method of the linear proportional transformation was used. Based on the correlation analysis in Section 3.1, it was found that grammage and thickness were positive indicators and the underfill factor was a negative indicator. Since there is an approximate exponential relationship between the air gap and the TPP value [23], the gap can be considered as a positive indicator. In the comprehensive evaluation of multiple indicators, the indicators must be homotrended, generally by converting the inverse and moderate indicators into positive indicators. Therefore, in this work, Equation (2) was used to dimensionlessize the positive indicators and Equation (3) was used to dimensionlessize the negative indicators. The results after dimensionless processing are shown in Table 3.

(2) $$x^{\prime }=\frac{x\;-\;\min(x)}{\max(x)\;-\;\min(x)}$$

(3) $$x^{\prime \prime }=\frac{\max(x)\;-\;x}{\max(x)\;-\;\min(x)}$$

The grey correlation coefficient can be calculated after using the following formula:

(4) $$\xi_{i}\left(k\right)=\frac{\underset{i}{min}\underset{k}{min}\left|x_{0}\left(k\right)-x_{i}\left(k\right)\right|+\rho \cdot \underset{i}{max}\underset{k}{max}\left|x_{0}\left(k\right)-x_{i}\left(k\right)\right|}{\left|x_{0}\left(k\right)-x_{i}\left(k\right)\right|+\varrho \cdot \underset{i}{max}\underset{k}{max}\left|x_{0}\left(k\right)-x_{i}\left(k\right)\right|}$$

where ρ is the identification coefficient (0 < ρ < 1). In practice, the identification coefficient is generally taken as ρ ≤ 0.5, which was taken as 0.5 in this work. $\underset{i}{min}\underset{k}{min}\left|x_{0}\left(k\right)-x_{i}\left(k\right)\right|$ is the minimum difference between the two levels; $\underset{i}{max}\underset{k}{max}\left|x_{0}\left(k\right)-x_{i}\left(k\right)\right|$ is the maximum difference between the two levels.

**Figure 5:**
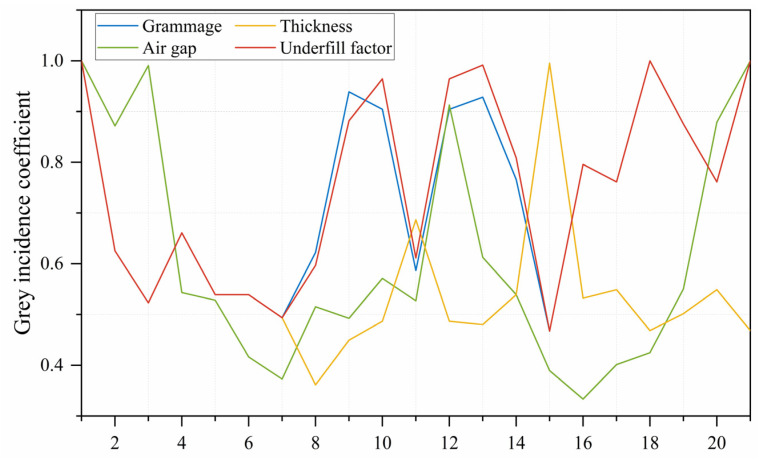
Correlation coefficient results.

Figure 5 shows the correlation coefficient results, from which it can be seen that the correlation coefficients for each influencing factor fluctuated between 0 and 1. This suggested that the judgement information is too scattered to allow for a comparative ranking of the influencing factors. To overcome this problem, the average of the correlation coefficients of the variables was calculated as a quantitative representation of the grey correlation grade. The formula for calculating the correlation grade is shown in Equation (5):

(5) $$r_{i}=\frac{1}{n}\sum_{k=1}^{n}\xi_{i}\left(k\right),\;k=1,\;2,\;\ldots ,\;n$$

where r_i_ is the grey correlation grade x_i_ to the reference series x_0_.

The grey correlation grade between the TPP value and the influencing factors were calculated and ranked according to the magnitude of the values, and the results are shown in Table 4. The grey correlation grade of all the influencing factors was greater than 0.5, indicating that the selected influencing factors were reasonable. Therefore, the four selected influencing factors can be used as independent variables in the multiple regression model to predict the TPP value. Furthermore, according to the ranking of the correlation grade between each factor and the TPP value, it can be seen that the influence degree on the TPP value was: underfill factor > grammage > air gap > thickness.

### 3.3. Regression Analysis and Prediction Models

#### 3.3.1. One-Dimensional Linear Regression Analysis

The analysis in Section 3.1 showed that there was a possible linear correlation between grammage, underfill factor, and TPP value. The relationship between grammage, underfill factor, and the dependent variable TPP value was therefore analyzed separately, using linear regression. The two one-dimensional linear regression equations established were:

(6) $$y_{1}=2.408x_{1}+119.84$$

(7) $$y_{2}=-133{.3x}_{4}+2579.7$$

where y_1_, y_2_ are predicted TPP values (kW∙s/m^2^), x_1_ is the grammage (g/m^2^), and x_4_ is the underfill factor.

**Figure 6:**
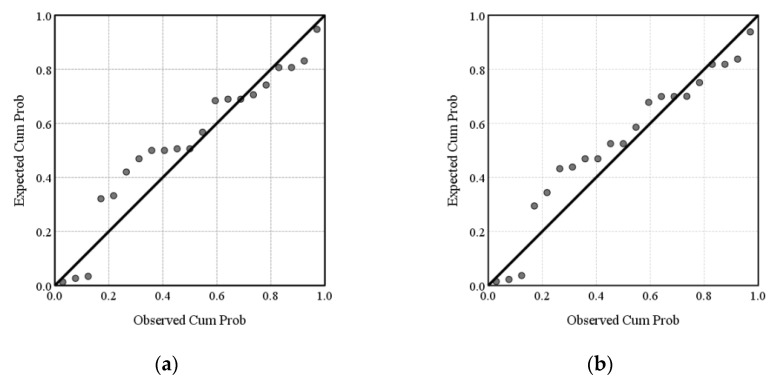
Normal P-P plots of grammage and underfill factor: (**a**) grammage; (**b**) underfill factor.

The coefficients in Equations (6) and (7) were obtained by fitting the data in Table 1 and Table 2. The resulting fitting equations were also tested by regression equation significance test (F-test), regression coefficient significance (*t*-test), and residual analysis. In the F-test, the result Significance F was obtained for the test. In the *t*-test, the result *p*-value was obtained and the decision was made by the *p*-value. In the F-test here, the significance F values for both Equations (6) and (7) were 0.000, and the significance F values for both equations were less than 0.01, indicating a significant linear relationship between the TPP value and both variables. The *t*-test yielded *p*-values of less than 0.01 for both the grammage and underfill factor, indicating that the effects of grammage and underfill factor on TPP value were significant. Figure 6a,b show normal P-P plots for the standardized residuals, where the scatter points of the standardized residuals were all distributed on or close to a straight line, so the residuals showed a normal distribution. The above significance test and residual analysis results indicated that it was reasonable to express the linear relationship between grammage, underfill factor, and TPP value using Equation (6) and Equation (7) respectively.

#### 3.3.2. Multiple Log-Linear Regression Analysis

There were linear relationships between grammage, underfill factor, and TPP value based on the results of Section 3.3.1. According to previous studies, there was a linear relationship between thickness and TPP value [5,11], and an approximate exponential relationship between air gap and TPP value [23]. For non-linear regression problems, it is difficult to calculate multivariate non-linear regression models directly. Therefore, when non-linear models (e.g., exponential models, etc.,) are encountered, they are usually converted to linear models for calculation. It was assumed here that the TPP value and the individual influencing factors were modelled as:

(8) $$y_{3}{=\beta }_{0}x_{1}^{\beta_{1}}e^{\beta_{2}x_{2}}x_{3}^{\beta_{3}}x_{4}^{\beta_{4}}$$

Taking logarithms on both sides of Equation (8) gave the following multiple linear regression model:

(9) $$\ln y_{3}=\beta_{0}+\beta_{1}\ln x_{1}+\beta_{2}x_{2}+\beta_{3}\ln x_{3}+\beta_{4}\ln x_{4}$$

where y_3_ is the predicted TPP value (kW∙s/m^2^), x_1_ is the grammage (g/m^2^), x_2_ is the air gap (mm), x_3_ is the thickness (mm), x_4_ is the underfill factor and β_0_, β_1_, β_2_, β_3_, and β_4_ are constants. The results of the measured values of each factor after taking the logarithm and the measured values of TPP are shown in Table 5.

The F-test and *t*-test were performed on Equation (9) using the data in Table 5 and the air gap (x_2_) data in Table 2. The coefficients in Equation (9) were also solved and the results were obtained as shown in Table 6. From Table 6, it can be seen that after the F-test, the model had a *p*-value of 0.000 (less than 0.01), indicating that there were independent variables in the model that would have an effect on the dependent variable. Lnx_1_, x_2_, and lnx_4_ had *p*-values less than 0.01, indicating that lnx_1_, x_2_, and lnx_4_ would have a significant effect on lny_3_. The *p*-value for lnx_3_ was 0.757 (>0.05), therefore the effect of lnx_3_ on lny_3_ was not significant and the variable could not be included in the regression model. The above analysis indicated that the model, Equation (9), was not valid and needed to be re-modelled. After re-modelling, the following equation were obtained:

(10) $$\ln y_{3}=\beta_{0}+\beta_{1}\ln x_{1}+\beta_{2}x_{2}+\beta_{4}\ln x_{4}$$

From Figure 2, the correlation coefficient between the grammage and the underfill factor was −1, indicating that there might be covariance between the two parameters. Table 7 was obtained after diagnosing the collinearity of Equation (10), and it was found that the variance inflation factor VIF value between the grammage and underfill factor was 1285.263 (>10) and the tolerance Tol was 0.001 (<0.1), so there was serious collinearity between the two parameters. Therefore, stepwise regression was used to remove the grammage or underfill factor to resolve the covariance of the model. After performing a stepwise regression, the grammage was removed, leaving the independent variables as the air gap and underfill factor. More specifically, the model can be expressed as:

(11) $$\ln y_{3}=\beta_{0}+\beta_{2}x_{2}+\beta_{4}\ln x_{4}$$

where x_2_ is the air gap (mm), x_4_ is the underfill factor, and y_3_ is the predicted TPP value (kW·s/m^2^).

The significance test of regression equation (Table 8, *p* = 0.000) showed a significant linear relationship between the TPP value and the air gap and the underfill factor. In the regression coefficient significance test, the *p*-value for the air gap was 0.001 and the *p*-value for the underfill factor was 0.000, both of which were less than 0.01, indicating that both the air gap and underfill factor had a significant effect on the TPP value. The goodness of fit test was also applied to the model, using the determination coefficient R^2^ and the adjusted R^2^ to determine the fit effect. The adjusted R^2^ for Equation (10) was found to be 0.880, indicating that the model fitted well. A residual independence test on the model yielded a result of 1.840 for the Debin–Watson test (D-W test), where the autocorrelation of the independent variables was not significant at D-W values close to 2, indicating that there was no interference between the sample data.

**Figure 7:**
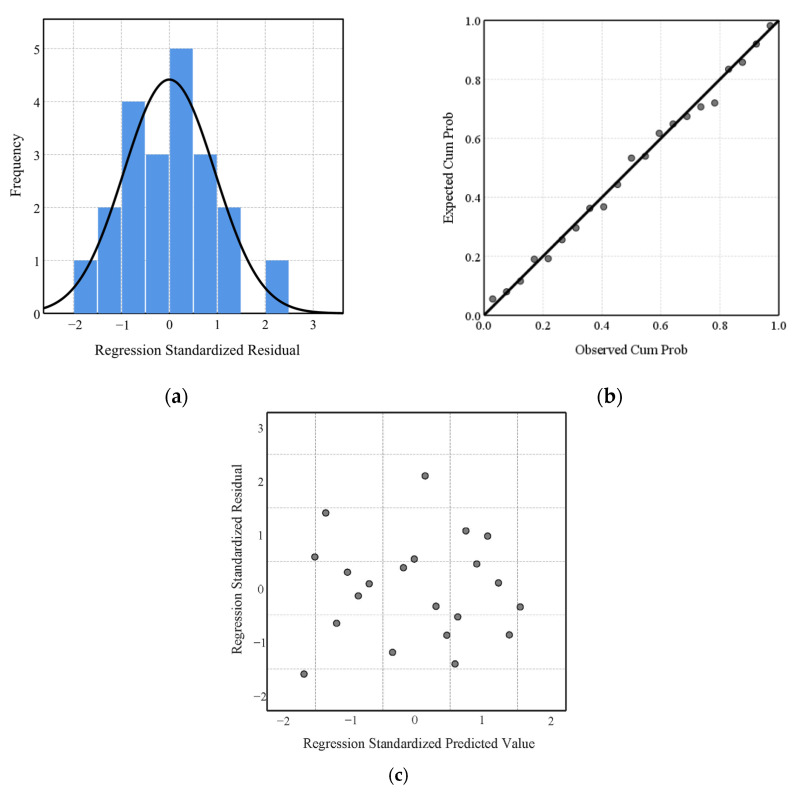
Standardized residual plot: (**a**) histogram; (**b**) P-P plot; (**c**) scatter plot.

Figure 7a shows a histogram of the standardized residuals, from which it can be seen that the standardized residuals of the regression and experimental values were approximately normally distributed. Figure 7b shows a normal probability plot (P-P plot) of the standardized residuals, where most of the scatter points of the standardized residuals were distributed on or close to the diagonal line, so the residuals were normally distributed. Figure 7c shows a scatter plot of the standardized residuals, in which the points were evenly distributed with no obvious regular variation, so the variance of the residuals was homogeneous. This result suggested that the relationship between the air gap, underfill factor, and TPP value was reasonably represented by the linear regression model of Equation (11).

#### 3.3.3. Multiple Non-Linear Regression Analysis

Based on the results of the stepwise regression, the independent variables were left with the air gap and underfill factor. A multiple non-linear regression model between the TPP value and the air gap and underfill factor can be derived by performing a multiple regression analysis from the data in Table 1 and Table 2 as follows.

(12) $$y_{4}=-89.701e^{-0.41467x_{2}}-133.27x_{4}+2597.10$$

where y_4_ is the predicted TPP value (kW∙s/m^2^), x_2_ is the air gap size (mm), and x_4_ is the underfill factor.

**Figure 8:**
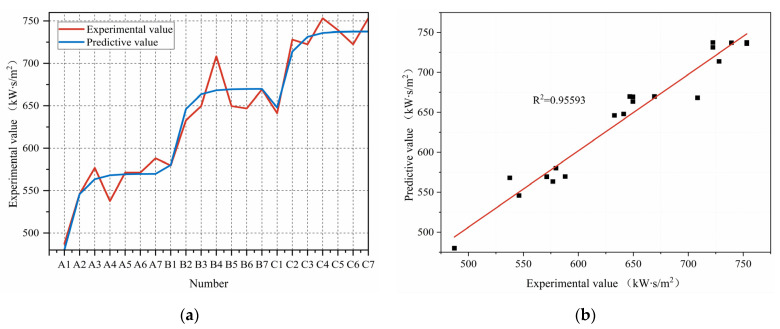
Line and contrast charts: (**a**) Line chart of measured TPP value and predicted TPP value; (**b**) comparison of measured TPP value and predicted TPP value.

Figure 8a,b show the measured TPP value versus the predicted TPP value, it can be seen that the predicted results agreed well with the measured results (R^2^ = 0.956, adjusted R^2^ = 0.948). Therefore Equation (12) can well predict the TPP value of Aramid 1414 for air gaps between 0 and 19.2 mm.

#### 3.3.4. Validation of Multiple Regression Prediction

The multiple regression model was applied to predict the TPP value of Aramid 1414 between the air gap of 0 and 19.2 mm, and the prediction results are shown in Table 9. The average relative errors of the two models were 3.31% and 1.94% respectively, indicating that the prediction accuracy of the multiple log-linear regression model was lower than that of the multiple non-linear regression model. Therefore, Equation (12) was a better predictor of the TPP value of Aramid 1414 between an air gap of 0 and 19.2 mm than Equation (11).

Therefore, the work carried out for the range of Aramid 1414 tested in this paper identified the key factors affecting the TPP value. The number of independent variables in the prediction model has been reduced, which is more conducive to the application of the model.

## 4. Conclusions

This work analyzed the correlation between grammage, thickness, air gap, underfill factor, and thermal protection performance, and established a multiple regression model. The following conclusions were obtained:

(1) By applying the grey correlation method, it was concluded that the influence degree on the TPP value is: underfill factor > grammage > gap > thickness.

(2) There is a positive correlation between grammage, air gap, and TPP value, with grammage having a significant positive correlation with TPP value. The underfill factor has a significant negative correlation with the TPP value.

(3) There is severe covariance between the grammage and underfill factor. The underfill factor was retained and the grammage was removed after a stepwise regression analysis was performed. An equation incorporating the air gap and underfill factor (Equation (12)) is developed to predict TPP value by multiple regression analysis.

(4) It is concluded that the non-linear regression model (Equation (12)) could better predict the TPP value of Aramid 1414 with air gaps between 0 and 19.2 mm, by comparing the TPP values predicted by the two multiple regression models. Moreover, the model (Equation (12)) needs to be tested for a wider range of fabrics and a wider range of parameters in future.

## Figures and Tables

**Table 1:** Physical properties of Aramid 1414 fabric specimens.

Composition	No.	Grammage (g/m^2^)	Thickness (mm)	Yarn Diameter (mm)	Coil Length (mm)	Underfill Factor
Aramid fiber 1414	A	180	0.85	0.25	3.80	15.21
	B	220	1.13	0.25	3.62	14.46
	C	250	1.01	0.25	3.48	13.95

**Table 2:** TPP values of fabric specimens.

No.	Gap Size (mm)	Second-Degree Burn Time (s)	TPP Value (kW∙s/m^2^)
A1	0	5.80	487.20
A2	3.2	6.50	546.00
A3	6.4	6.90	576.80
A4	9.6	6.40	537.60
A5	12.4	6.80	571.20
A6	16	6.80	571.20
A7	19.2	7.00	588.00
B1	0	6.90	579.60
B2	3.2	7.50	632.80
B3	6.4	7.70	649.60
B4	9.6	8.43	708.40
B5	12.4	7.73	649.60
B6	16	7.70	646.80
B7	19.2	7.97	669.20
C1	0	7.63	641.20
C2	3.2	8.67	728.00
C3	6.4	8.60	722.40
C4	9.6	8.97	753.20
C5	12.4	8.80	739.20
C6	16	8.60	722.40
C7	19.2	8.97	753.20

**Table 3:** Dimensionless results for TPP value and its influencing factors.

TPP Value (x_0_)	Grammage (x_1_)	Air Gap (x_2_)	Thickness (x_3_)	Underfill Factor (x_4_)
0.00000	0.00000	0.00000	0.00000	0.00000
0.22105	0.00000	0.16667	0.00000	0.00000
0.33684	0.00000	0.33333	0.00000	0.00000
0.18947	0.00000	0.50000	0.00000	0.00000
0.31579	0.00000	0.64583	0.00000	0.00000
0.31579	0.00000	0.83333	0.00000	0.00000
0.37895	0.00000	1.00000	0.00000	0.00000
0.34737	0.57143	0.00000	1.00000	0.59685
0.54737	0.57143	0.16667	1.00000	0.59685
0.61053	0.57143	0.33333	1.00000	0.59685
0.83158	0.57143	0.50000	1.00000	0.59685
0.61053	0.57143	0.64583	1.00000	0.59685
0.60000	0.57143	0.83333	1.00000	0.59685
0.68421	0.57143	1.00000	1.00000	0.59685
0.57895	1.00000	0.00000	0.58071	1.00000
0.90526	1.00000	0.16667	0.58071	1.00000
0.88421	1.00000	0.33333	0.58071	1.00000
1.00000	1.00000	0.50000	0.58071	1.00000
0.94737	1.00000	0.64583	0.58071	1.00000
0.88421	1.00000	0.83333	0.58071	1.00000
1.00000	1.00000	1.00000	0.58071	1.00000

**Table 4:** Grey correlation coefficients and correlations of influencing factors.

No.	Grammage (x_1_)	Air Gap (x_2_)	Thickness (x_3_)	Underfill Factor (x_4_)
A1	1.00000	1.00000	1.00000	1.00000
A2	0.62556	0.87165	0.62556	0.62556
A3	0.52298	0.99058	0.52298	0.52298
A4	0.66091	0.54322	0.66091	0.66091
A5	0.53905	0.52807	0.53905	0.53905
A6	0.53905	0.41642	0.53905	0.53905
A7	0.49355	0.37290	0.49355	0.49355
B1	0.62238	0.51530	0.36137	0.59682
B2	0.93883	0.49240	0.44930	0.88185
B3	0.90426	0.57123	0.48671	0.96427
B4	0.58670	0.52691	0.68679	0.61139
B5	0.90426	0.91275	0.48671	0.96427
B6	0.92819	0.61281	0.48004	0.99153
B7	0.76605	0.53905	0.53905	0.80869
C1	0.46726	0.38945	0.99526	0.46726
C2	0.79583	0.33333	0.53224	0.79583
C3	0.76130	0.40133	0.54890	0.76130
C4	1.00000	0.42482	0.46830	1.00000
C5	0.87526	0.55050	0.50179	0.87526
C6	0.76130	0.87891	0.54890	0.76130
C7	1.00000	1.00000	0.46830	1.00000
Correlation grade	0.74727	0.61293	0.56832	0.75528
Rank order	2	3	4	1

**Table 5:** The influence factor value and TPP value after taking the logarithm.

lnx_1_	x_2_	lnx_3_	lnx_4_	lny_3_
5.1930	0	−0.15689	2.7222	6.1887
5.1930	3.2	−0.15689	2.7222	6.3026
5.1930	6.4	−0.15689	2.7222	6.3575
5.1930	9.6	−0.15689	2.7222	6.2871
5.1930	12.4	−0.15689	2.7222	6.3477
5.1930	16	−0.15689	2.7222	6.3477
5.1930	19.2	−0.15689	2.7222	6.3767
5.3936	0	0.12737	2.6715	6.3623
5.3936	3.2	0.12737	2.6715	6.4502
5.3936	6.4	0.12737	2.6715	6.4764
5.3936	9.6	0.12737	2.6715	6.5630
5.3936	12.4	0.12737	2.6715	6.4764
5.3936	16	0.12737	2.6715	6.4720
5.3936	19.2	0.12737	2.6715	6.5061
5.5215	0	0.01784	2.6357	6.4633
5.5215	3.2	0.01784	2.6357	6.5903
5.5215	6.4	0.01784	2.6357	6.5826
5.5215	9.6	0.01784	2.6357	6.6243
5.5215	12.4	0.01784	2.6357	6.6056
5.5215	16	0.01784	2.6357	6.5826
5.5215	19.2	0.01784	2.6357	6.6243

**Table 6:** Regression analysis results.

		Regression Coefficient	t	Significance (*p*)
(Constant)		0.167	6.545	0.000 **
Grammage	lnx_1_	0.945	14.245	0.000 **
Air gap	x_2_	0.006	3.900	0.000 **
Thickness	lnx_3_	−0.038	−0.310	0.757
Underfill factor	lnx_4_	0.432	3.489	0.000 **
R^2^		0.892		
Adjusted R^2^		0.873		
F		F = 171,060.341, *p* = 0.000		

** *p* ≤ 0.01.

**Table 7:** Collinearity statistics.

Model		t	*p*	Collinearity Statistics	
				Tolerance	VIF
1	(Constant)	0.331	0.744	N/A	N/A
	Grammage	0.032	0.975	0.001	1285.263
	Air gap	3.925	0.001	1.000	1.000
	Underfill factor	−0.280	0.783	0.001	1285.263

**Table 8:** Analysis of test results.

Model		Coefficient	t	*p*	Collinearity Statistics	
					Tolerance	VIF
1	(Constant)	14.648	20.440	0.000	N/A	N/A
	Air gap	0.006	4.036	0.001	1	1
	Underfill factor	−3.082	−11.514	0.000	1	1
D-W value		1.840				
Adjusted R^2^		0.880				

**Table 9:** Predicted TPP value of Aramid 1414 with gap size between 0 and 19.2 mm.

No.	Multiple Logarithmic Regression Models		Multiple Nonlinear Regression Models		Measured TPP Value
	Predicted Value	Relative Error	Predicted Value	Relative Error	
A1	522.3	7.20%	480.4	1.40%	487.2
A2	532.4	2.49%	546.3	0.05%	546.0
A3	542.7	5.91%	563.8	2.25%	576.8
A4	553.2	2.90%	568.4	5.73%	537.6
A5	562.6	1.51%	569.5	0.30%	571.2
A6	574.9	0.65%	569.9	0.23%	571.2
A7	586.0	0.34%	570.0	3.06%	588.0
B1	610.6	5.35%	580.3	0.12%	579.6
B2	622.4	1.64%	646.2	2.12%	632.8
B3	634.5	2.32%	663.7	2.17%	649.6
B4	646.8	8.70%	668.3	5.66%	708.4
B5	657.8	1.26%	669.5	3.06%	649.6
B6	672.1	3.91%	669.9	3.57%	646.8
B7	685.2	2.39%	670.0	0.12%	669.2
C1	681.8	6.33%	648.3	1.11%	641.2
C2	695.0	4.53%	714.2	1.90%	728.0
C3	708.5	1.92%	731.7	1.29%	722.4
C4	722.2	4.12%	736.3	2.24%	753.2
C5	734.4	0.65%	737.5	0.23%	739.2
C6	750.5	3.89%	737.9	2.15%	722.4
C7	765.0	1.57%	738.0	2.02%	753.2
Average value	-	3.31%	-	1.94%	-

## Data Availability

All data, results, and models used or generated during the study appear in the submitted article.
